# A point-of-use drinking water quality dataset from fieldwork in Detroit, Michigan

**DOI:** 10.1038/s41597-024-03298-w

**Published:** 2024-05-03

**Authors:** Alyssa Schubert, Jacob Harrison, Linda Kent-Buchanan, Victor Bonds, Shawn P. McElmurry, Nancy G. Love

**Affiliations:** 1 University of Michigan, Department of Civil and Environmental Engineering, Ann Arbor, USA; 2 Detroit Community Researcher, Detroit, USA; 3 Wayne State University, Department of Civil and Environmental Engineering, Detroit, USA

**Keywords:** Environmental monitoring, Water resources

## Abstract

Drinking water quality sensor technology has rapidly advanced, facilitating the collection of rich datasets and real-time analytics. However, sensors have not yet been widely applied to monitor drinking water quality in premise plumbing. Richer quality of data in premise plumbing offers an improved understanding of the quality of drinking water present at the point-of-use. In this paper, online drinking water quality sensor nodes were temporarily installed in twenty-four homes in Detroit, Michigan. The water quality sensor nodes took measurements of five drinking water quality parameters every five minutes for four weeks. Additionally, free chlorine and lead were sampled periodically within each home. Together, these data make up a dataset that captures drinking water quality over time in a legacy city with an oversized drinking water system. This dataset offers more frequent measurements amongst more sample homes than are typically available in premise plumbing or at the tap. The data can be used to investigate temporal trends in drinking water quality, including diurnal patterns and anomaly detection. Additionally, this dataset could be utilized to evaluate water quality in comparison with other cities.

## Background & Summary

Regulatory monitoring for drinking water quality primarily occurs at the drinking water treatment plant or within the water distribution system, the network of pipes that conveys drinking water from the treatment plant to the service line. Beyond the service line, drinking water is conveyed to the point-of-use, e.g., the tap, in buildings or homes through a series of pipes called premise plumbing. While there are some exceptions, for example, the Lead and Copper Rule, which requires collecting drinking water samples from taps within homes, drinking water quality is not extensively monitored beyond the service line^1^. However, a variety of physicochemical and biological changes can occur as drinking water travels from the service line into premise plumbing and to the point-of-use. For example, premise plumbing pipes have a small diameter, which can lead to higher residence times as well as higher rates of chlorine decay and microbiological growth^2–4^. Thus, there is a growing interest in understanding water quality in premise plumbing motivated by the desire to improve the management of drinking water in homes and buildings^5,6^. As developments of automated technology, such as online water quality sensors, continue to advance, there is more opportunity for real-time monitoring in premise plumbing to close the knowledge gaps about urban drinking water quality management^2,7^. More resolved data in premise plumbing provides the opportunity for water service providers, water users, and policymakers to better understand the dynamics of water quality and water user exposure at the point-of-use. For example, a better understanding of premise plumbing water quality can help inform guidance around how frequently, and with what density, monitoring in premise plumbing should occur to capture significant trends or changes to water quality or exposure to contaminants at the point-of-use. Further, premise plumbing water quality data can be used to make more informed decisions about upstream water treatment. For example, water distribution systems in legacy cities such as Detroit may experience challenges in maintaining adequate chlorine residual. If chlorine residual is very low in monitored premise plumbing locations, additional actions, such as boosting chlorine upstream, could be taken. Moreover, resolved data about water quality in premise plumbing opens the opportunity to provide more actionable information to water users. Actionable information is information that is substantial, relevant, and supports one’s self-efficacy and decision-making^8–10^. Furthermore, individualized water data also provides information that can hold utilities accountable. While public drinking water systems are required to release an annual Consumer Confidence Report (CCR), the data in CCRs are highly summarized and hard to understand for water users^11–13^. Data collected in premise plumbing may be more actionable to water users than data summarized at the system-scale^2,7^.

This dataset was generated as the result of a study designed to monitor drinking water quality in Detroit, Michigan homes, using twelve online, autonomous water quality sensor nodes. The data were used to examine the effect of individualized drinking water quality data on water user perceptions of drinking water quality and the water service provider^14^. At the time of submission, a related paper utilizing the data to model drinking water quality in premise plumbing was also under development. The following parameters were monitored using the drinking water quality sensor node: electrical conductivity (EC, μs/cm), oxidation-reduction potential (ORP, mV), pH, temperature (°C), and pressure (PSI). Electrical conductivity is an indicator of the total dissolved solids (TDS) and salinity of drinking water. High TDS can affect the taste, sight, and smell of drinking water, and can influence one’s willingness to use the water. ORP is a measure of the oxidizing or reducing conditions of an environment and is often used as an indicator for the presence of disinfectant in an aqueous system. ORP measurements also provide information about the appropriateness of the environment for microbial growth and offer an indication of the likelihood of pipe corrosion^15,16^. Data from pH measurements offer information about the acidity of the aquatic environment, including the effective range of a disinfectant and the potential for corrosion to occur. For instance, decreasing pH can increase metal solubility, which is of interest for understanding the likelihood of lead exposure in drinking water^17^. Water temperature fluctuates seasonally and can serve as an indicator of microbiological stability. For example, rates of microbiological growth and chemical reactions, such as chlorine decay, are influenced by water temperature^18^. Higher water temperatures have been documented to increase the growth of certain opportunistic pathogens^19,20^ or indicator organisms, such as coliforms^19^. Further, water temperature can affect how willing water users are to drink the water. Lastly, water pressure can provide information about the infrastructure status. For example, low or intermittent water pressure can indicate the presence of a leak.

In addition to the parameters measured by the sensor nodes, free chlorine and lead were measured via supplemental grab samples. Free chlorine is the disinfectant used in the Detroit water system. Free chlorine residual is monitored in drinking water systems to ensure there is enough disinfectant to inhibit microbiological regrowth or recontamination as water is conveyed from the treatment plant to the point-of-use. Lead is a heavy metal that is primarily present in drinking water via corrosion from lead-containing plumbing materials and can cause negative health effects when ingested as stated by the Centers for Disease Control and Prevention (https://www.cdc.gov/nceh/lead/prevention/sources/water.htm). In this study, the first- and fifth-liters of drinking water from the tap after a period of stagnation (>8 hours) were sampled. The first-liter can be assumed to be more representative of the water in household fixtures, whereas the fifth-liter can be assumed to be more representative of the water in the service line, which conveys drinking water from the water main to the home, and may contain lead^21^.

The data presented in this paper provide a snapshot of high-resolution drinking water quality from within twenty-four homes in a legacy city with an oversized water distribution system. The sensor node data can be used to explore temporal relationships with drinking water quality, including diurnal trends and anomaly detection. Further, the additional grab sample data may be used to better understand the behaviors of free chlorine and lead at the point-of-use.

## Methods

Twelve online water quality sensor nodes were deployed in premise plumbing in twenty-four participant homes for approximately one month each in a Detroit, Michigan community. Prior to beginning recruitment for participants, a study protocol was developed and reviewed by the Institutional Review Board at the University of Michigan (HUM 00199905) and Wayne State University (IRB 21-07-3786). Both IRBs were declared ‘exempt.’ Recruitment for participants began in summer 2021 at a local farmers market. In early 2022, two community members were hired to serve as community researchers and team liaisons. The community researchers continued recruitment at local events and through word-of-mouth. All interested participants reviewed a written informed consent during an initial visit to their home, which reviewed the benefits and risks of the study and emphasized that participants could leave the study at any time. Signatures were not required because the study was considered exempt. More detailed information about participant recruitment, involvement, demographic information, and compensation is available in Schubert *et al*.^14^. Deployments spanned from March to December 2022. The sensor nodes required a nearby outlet for power and used a Particle Photon or Boron to convey data to an online cloud server, using either a WI-FI connection or a cellular network, respectively. The sensor nodes were placed in the home depending on the location of the nearby outlet and the preference of the resident, which could include the following locations: the kitchen sink, the bathroom sink, the toilet, the utility sink, or the laundry machine. The water quality sensor nodes contained a pressure probe, temperature probe, ORP probe, EC probe, and pH probe. A solenoid valve was used to control the flow through the sensor node cell. The blueprints for building and operating the sensor nodes are open source and can be found in more detail in Martinez-Paz *et al*.^7^.

**Fig. 1:**
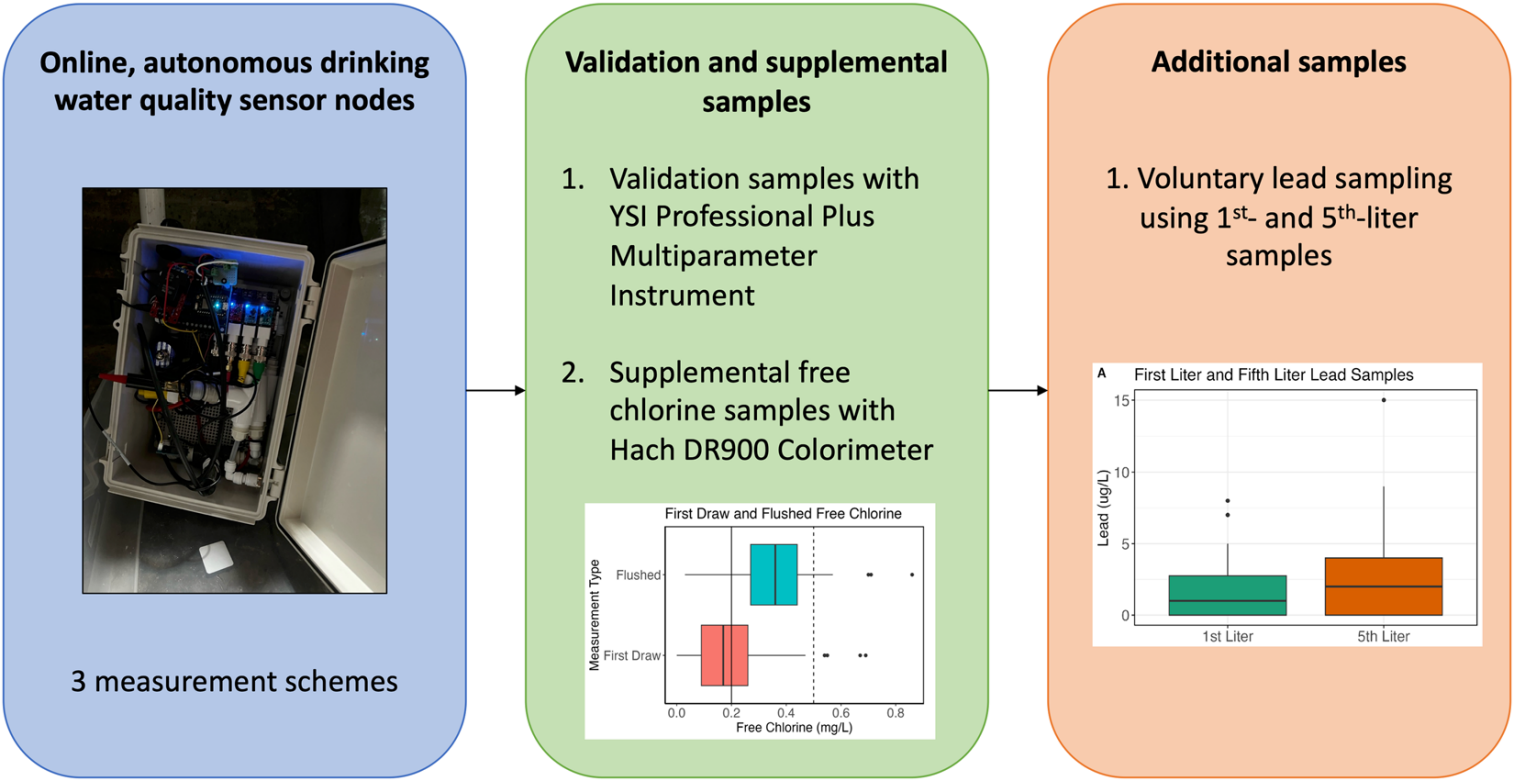
Schematic displaying the different measurement schemes that were utilized to collect the water quality sensor node data.

Three measurement schemes were adopted across the sensor nodes (Fig. 1). In the first scheme, the sensor node took a pressure, temperature, pH, EC, and ORP reading every 5 minutes. Every twelfth measurement period, or one hour, the solenoid valve opened and allowed new water to flow through the cell for five seconds. Therefore, only the measurements taken every hour were representative of water that had just been drawn in from the sensor node inlet port. Five sensor nodes, identified with “S” as the prefix, (S58, S74, S21, S85, and S46) were programmed with this scheme. The second scheme is the same as the first with the addition of two programmed five-minute flushes, during which the valve opened for five minutes, at 4:00AM and 4:00PM (n = 10, S15, S59, S88, S90, S35, S75, S84, S12, S74, S95). The flush times were chosen to capture the effects of flushing after extended periods of stagnation: overnight after sleeping (4:00AM) and during the day before residents came home from work (4:00PM). In the third scheme, the solenoid valve opened every five minutes for five seconds, allowing new water to enter the flow cell every five minutes. Additionally, there was a five-minute flush at 4:00PM and 4:00AM (n = 9, S14, S93, S31, S7, S69, S16, S53, S48, S10).

**Fig. 2:**
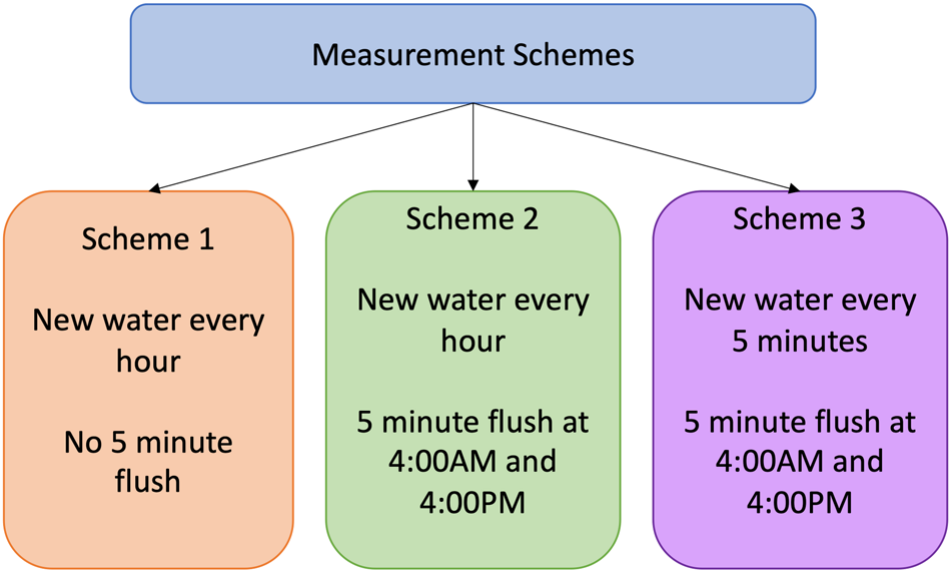
Data collection methods and types. Data were collected using drinking water quality sensor nodes (left box), grab samples to validate and supplement the sensor node data (middle box), and 1^st^- and 5^th^-liter samples for lead analysis (right box).

Two types of grab samples, ‘first draw’ and ‘flushed,’ were taken in participant homes to validate or supplement the sensor node data. First draw samples were taken from the tap immediately after it was turned on by a study team member. Flushed samples were taken from the tap after allowing water to run for five minutes. To validate the sensor node data, measurements of ORP, EC, pH, and temperature were taken using a YSI Professional Plus Multiparameter instrument (Yellow Springs, Ohio, USA). The YSI instrument was calibrated every morning before use. To supplement the sensor node data, free chlorine measurements were taken using a Hach DR900 (Loveland, Colorado, USA) at the same time as the validation measurements. The validation and free chlorine first draw and flushed measurements were taken three times for each participant. The samplings occurred when the sensor node was installed, at a mid-study check in, and when the sensor was un-installed. Lastly, for all study participants that elected to participate, lead measurements were taken. Participants were instructed not to use any water in the home for at least eight hours prior to the sample collection. In accordance with the revised Lead and Copper Rule for Michigan (The Michigan Safe Drinking Water Act, 1976 PA 399), participants used sterile sample bottles to collect the first- and fifth-liter of water from their kitchen faucet after the stagnation period^21^. The lead samples were delivered to a certified laboratory for analysis within twenty-four hours of sample collection. Samples were preserved with nitric acid until analyzed. Results were returned within one week of sample collection. Figure 2 shows a summary of the data collected and the method of measurement.

## Data Records

All data records are available in Figshare^22^. The Excel spreadsheet titled “Lead, chlorine, and home data” contains the lead, chlorine, and self-reported home age data for each study participant in separate sheets. There are twenty-four Excel spreadsheets, one per participant, containing raw, uncorrected sensor node data. These spreadsheets have a “S” prefix. The Excel spreadsheet titled “YSI data” contains the validation grab samples taken at each home.

**Fig. 3:**
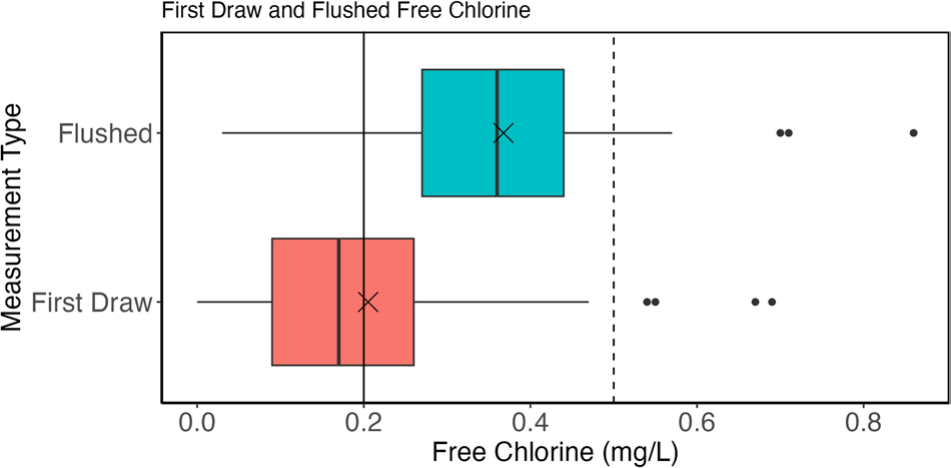
Boxplots of flushed (blue) and first draw (red) free chlorine samples for the twenty-four homes included in the study. Means are denoted by a black X.

**Fig. 4:**
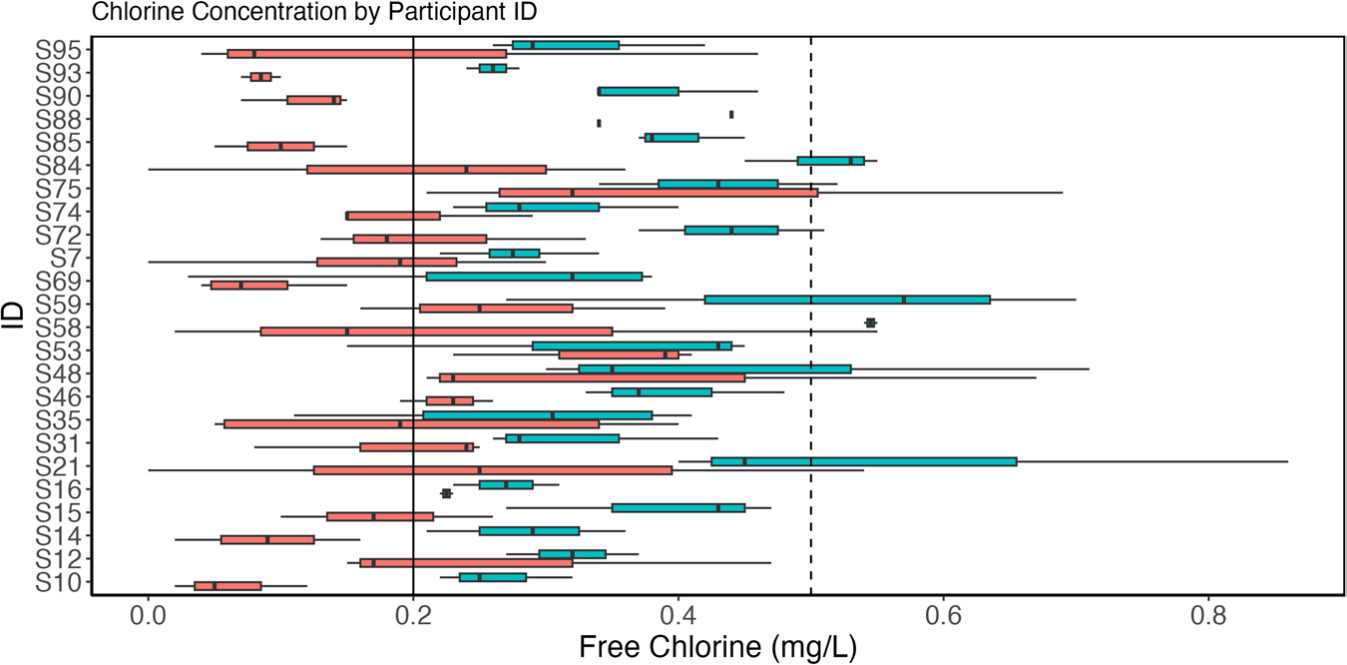
Boxplots of flushed (blue) and first draw (red) free chlorine samples per ID, or participant home.

Figures 3 and 4 provide an overview of the free chlorine data. The solid vertical line on both figures denotes a free chlorine concentration of 0.2 mg/L; concentrations less than this are associated with higher microbiological growth^23^. The dashed vertical line marks a free chlorine concentration of 0.5 mg/L, which is a common operating minimum^24^. The average, minimum, and maximum free chlorine residual concentrations measured across all homes for first draw samples were 0.20 mg/L, non-detect, and 0.69 mg/L, respectively (Fig. 3). For flushed samples, the average, minimum, and maximum were 0.37 mg/L, 0.03 mg/L, and 0.86 mg/L. Note that the Hach DR900 has a detection limit of 0.02 mg/L according to the manufacturer. In almost all sampling cases across all study homes, free chlorine residual increased after a five-minute flush, as evidenced by the shift seen in boxplot spreads in Figs 3 and 4. This increase was anticipated under the assumption that the five-minute flush was drawing fresher drinking water from the distribution system with a higher chlorine residual into the premise plumbing.

**Fig. 5:**
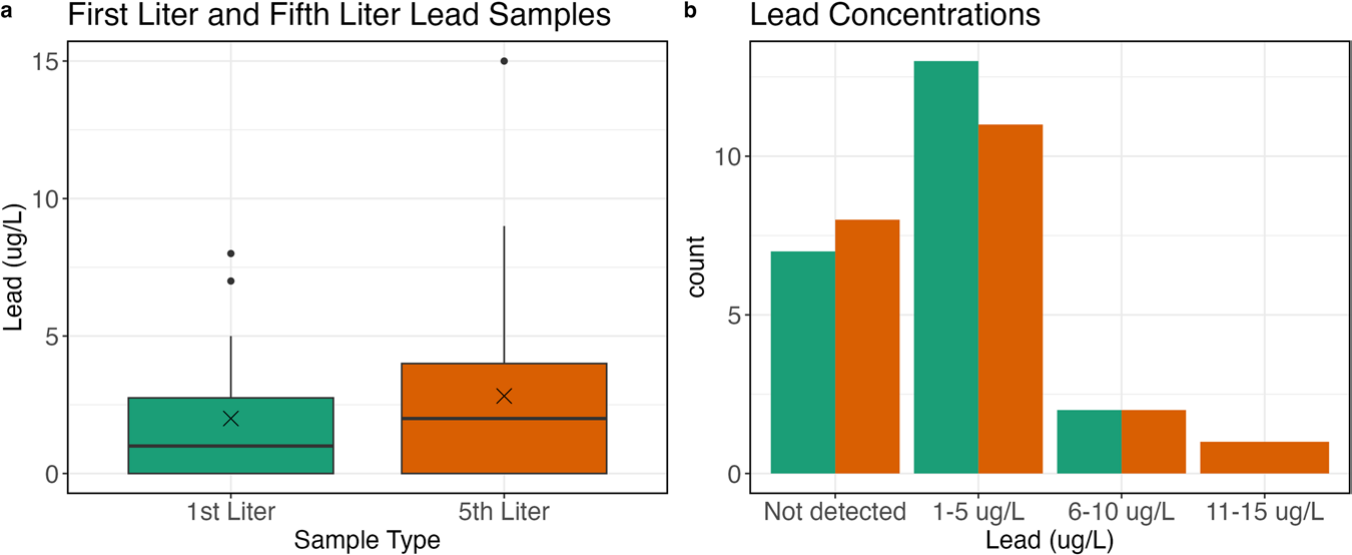
(**a**) shows boxplots of first-liter (green) and fifth-liter (orange) samples. Means are denoted by a black X. (**b**) shows the counts of samples that were non-detect, between 1 and 5 ug/L, 6 and 10 ug/L, and 11 and 15 ug/L.

Figure 5 provides an overview of the lead data. Out of the 24 study participants, 22 elected to participate in lead sampling. Within the study homes, the range of samples was between 0 (not detected, <0.5 μg/L) and 15 μg/L. The 90^th^ percentile observed in homes during this study was 5.7 μg/L. Figure 5a provides boxplots for the first- and fifth-liter lead concentrations from the study samples. The median lead concentrations in the first- and fifth-liter were 1 μg/L and 2 μg/L, respectively. In eight of the sampled study homes, the fifth-liter lead concentration was greater than the first-liter, suggesting that there may be a lead service line present in these homes. Seven of the homes had a greater concentration of lead in the first-liter than the fifth-liter. Figure 5b provides the counts of samples that are not detected, between 1 and 5 μg/L, 6 and 10 ug/L, and 11 to 15 μg/L. Most homes had lead concentrations ranging from 1 to 14 μg/L. One reason for the high counts of detected lead may be the age of the homes or pipes. The use of lead pipes, solder, and flux was not banned until 1986^25^. While we did not determine pipe age in each participant home, we did collect information on the home age. The home ages most frequently reported by participants were between 1900 to 1930 (54%). No homes participating in the study were built later than 1980.

## Technical Validation

The drinking water quality sensor probes were regularly examined and recalibrated after every deployment. If calibration measurements were not as expected or unstable, the probe underwent a troubleshooting process, beginning with checking probe connections and escalating up to probe replacement. Uncertainties in the water quality sensor probe data may arise from measurement errors such as drift. In particular, ORP probes have been shown to drift or provide inconsistent measurements from instrument to instrument^15^. The usage notes contain suggested approaches for cleaning the sensor node data to address this uncertainty.

## Usage Notes

In sensor placement optimization literature, sensors may be assumed to be ‘perfect’ for model simplicity^26,27^. However, in reality, sensors are prone to errors, including drift, which causes the reported parameter measurements to gradually move farther away from the true values^2,28,29^. Consequently, we suggest two downstream data cleaning steps that may be applied to the sensor node data to ensure that the data are accurate and reliable. Accuracy and reliability of the data are important for both utilities that may use the data to make decisions about drinking water management, and for water users or researchers who may have access to a subset of the water quality data. Users of this dataset should be aware that there are some instances of missing data in the sensor node datasets. Occasionally, we experienced the sensor nodes requiring a ‘warm up period,’ during which the probes were online for a few minutes before taking measurements. Further, there are missing data from events that when a probe was disconnected or malfunctioned. These incidents were infrequent.

**Fig. 6:**
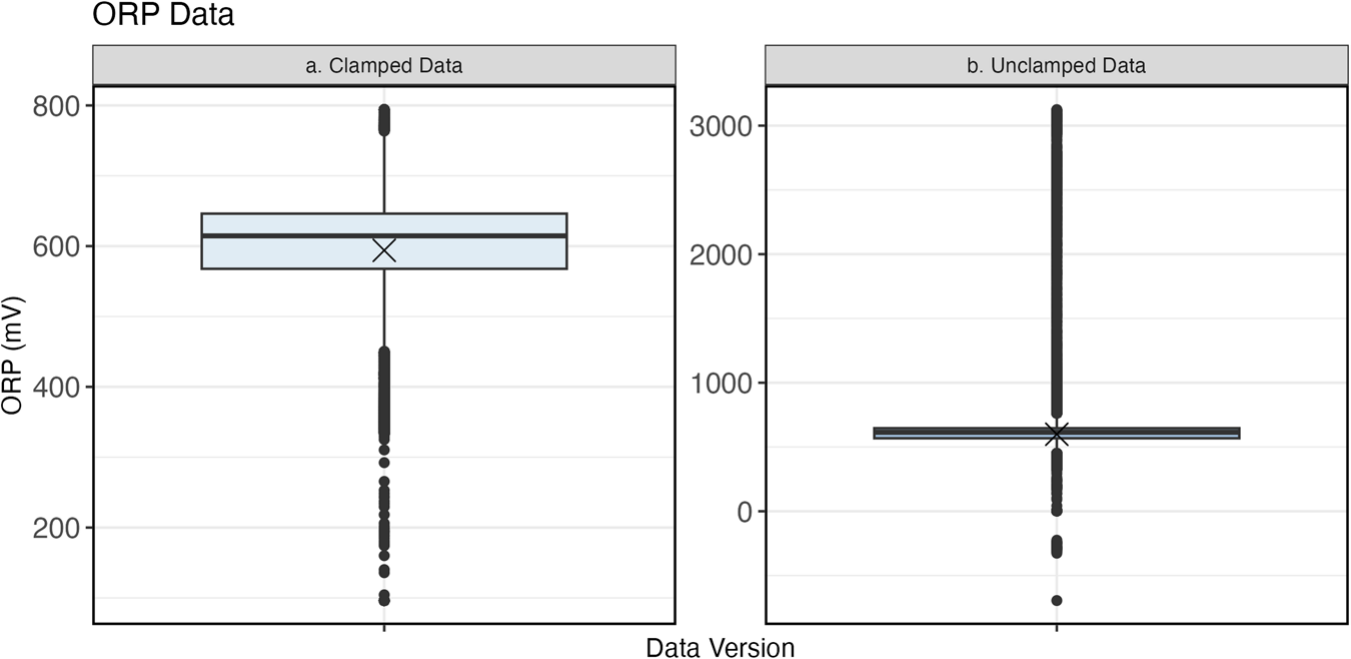
(**a**) shows the spread of clamped ORP data. (**b**) shows the spread of unclamped ORP data, with a much wider y-axis. Means are denoted by a black X.

The two suggested downstream data cleaning steps include correction and clamping. First, sensor node data can be corrected based on the YSI validation measurements, which are considered to be more accurate than the sensor node data because the YSI probe is more frequently calibrated. In one out of the 24 participants, a validation measurement was not recorded for a participant while in the field due to user error; otherwise, all participants have at least three validation measurements on which to correct the sensor node data. A number of methods can be used to develop a correction coefficient for the sensor node data. As a test case, we developed a linear relationship between the sensor node data and YSI validation measurements for each home. Second, to address outliers and minimize the effect of any drifting that may have occurred, the sensor node data can be clamped. Clamping is a useful method for data cleaning that replaces extreme values with an upper and lower limit value, or a clamping bound. Clamping allows outliers to be addressed while retaining all rows of data and can be a helpful approach for minimizing the presence of extreme values in the maximums and minimums of the corrected, but unclamped, data. Two clamping bounds, a minimum and maximum, for each measured parameter can be developed based on the YSI validation measurements. We suggest using the maximum and minimum parameter value measured by the YSI validation measurements, minus or plus ten percent of those values, respectively. Figure 6 demonstrates an example whereby this approach was applied to the ORP data, which had a wide range of values that were not physically expected in drinking water systems including negative and extreme positive values. Notably, the range of measured values for the clamped data (Fig. 6a) is smaller compared to the unclamped data (Fig. 6b). For example, unclamped ORP values range from −695 mV to 3,125 mV, which is unreasonable in drinking water environments^23^. The clamped data range from 96.3 mV to 794 mV. These bounds were determined as a conservative minimum and maximum based on the YSI validation sample measurements.

The data presented in this paper demonstrate the variability in chlorine residual and lead concentrations, among other drinking water quality parameters, across homes in an urban legacy drinking water distribution system. Future work using this dataset can explore the significance of temporal and spatial trends in the water quality data collected by the sensor nodes within and across homes to better understand the dynamics of water quality in premise plumbing. For example, this dataset spans across seasons and can be used to investigate the impact of seasonal effects on the measured water quality parameters. Further, while the dataset described in this study does not include the spatial coordinates of the homes monitored in order to anonymize study participants, this dataset could be used at the city-scale to compare drinking water quality between cities with similar or dissimilar demographics, water distribution sizing, or water chemistry. Future research in this area should help identify how many sensor nodes should be placed, and in which premise plumbing locations, for optimal cost/benefit trade-offs. The data collected by future research around premise plumbing water quality can be used to develop guidelines for water quality management in buildings and homes, including best practices for building residents, such as flushing, and actions that can be taken by the water service provider, such as chlorine boosting. Finally, future research should further explore the spatiotemporal relationship of water quality at the point-of-use in the context of household-scale water insecurity, housing affordability, and socioeconomic factors.

## Data Availability

Chlorine and lead data were processed in Excel. The test case for the suggested data correction approach was completed in Excel. The code for the suggested data clamping approach was written in R and is available in the Figshare repository.
